# Zinc disrupts central carbon metabolism and capsule biosynthesis in *Streptococcus pyogenes*

**DOI:** 10.1038/srep10799

**Published:** 2015-06-01

**Authors:** Cheryl-lynn Y. Ong, Mark J. Walker, Alastair G. McEwan

**Affiliations:** 1 School of Chemistry and Molecular Biosciences and Australian Infectious Diseases Research Centre, The University of Queensland, St. Lucia 4072, Australia

## Abstract

Neutrophils release free zinc to eliminate the phagocytosed bacterial pathogen *Streptococcus pyogenes* (Group A *Streptococcus*; GAS). In this study, we investigated the mechanisms underpinning zinc toxicity towards this human pathogen, responsible for diseases ranging from pharyngitis and impetigo, to severe invasive infections. Using the globally-disseminated M1T1 GAS strain, we demonstrate that zinc stress impairs glucose metabolism through the inhibition of the glycolytic enzymes phosphofructokinase and glyceraldehyde-3-phosphate dehydrogenase. In the presence of zinc, a metabolic shift to the tagatose-6-phosphate pathway allows conversion of D-galactose to dihydroxyacetone phosphate and glyceraldehyde phosphate, partially bypassing impaired glycolytic enzymes to generate pyruvate. Additionally, zinc inhibition of phosphoglucomutase results in decreased capsule biosynthesis. These data indicate that zinc exerts it toxicity via mechanisms that inhibit both GAS central carbon metabolism and virulence pathways.

Zinc is a significant factor in the innate immune defence against pathogens^1^. This metal ion is an absolute nutritional requirement for the growth of all cells and it is essential for the activity of a wide variety of enzymes^2^. The ability of the host to withhold zinc from the bacterial pathogen is an important aspect of nutritional immunity and it is exemplified by the action of the human protein calprotectin^3^,^4^. Calprotectin has a high affinity for zinc and the sequestration of this critical metal ion leads to starvation of the bacterial pathogen^5^. Nonetheless, it is also established that zinc deficiency in humans is linked to increased susceptibility to bacterial infections^6^. This increased susceptibility has been ascribed to a decrease in the capability of the innate immune system as a consequence of zinc limitation^6^. Altered levels of zinc have been measured during inflammation and high levels of zinc result in the activation of various immune cells, leading to the suggestion that zinc may have an additional signalling role in relation to host defence against pathogens^7^.

Approximately 15% of zinc has been described as labile (readily available) in the airway mucosal epithelium^8^ and also specific sites of infection where recruitment of innate immune cells can result in an influx of zinc^9^,^10^. Recently, in a landmark study, Botella and co-workers demonstrated that zinc is trafficked to the phagosome of macrophages infected with *Mycobacterium tuberculosis*, indicating that elevated zinc may play a more direct antimicrobial role^9^. Subsequently, it has been demonstrated that internalization of *Streptococcus pyogenes* (Group A *Streptococcus*; GAS) triggers the release of zinc within human neutrophils^10^. The zinc-specific cation diffusion facilitator, CzcD, was found essential for bacterial resistance against zinc-mediated killing by human neutrophils^10^. Furthermore, deletion of *czcD* resulted in reduced virulence in a murine infection model of GAS invasive disease^10^. These data are consistent with a direct antimicrobial effect of zinc on bacteria during infection.

How might zinc exert an antimicrobial effect? Recently, McDevitt and co-workers showed that in *Streptococcus pneumomoniae*, zinc prevents the uptake of manganese by binding irreversibly to the high affinity manganese solute binding protein PsaA^11^,^12^. As a consequence, *S. pneumoniae* becomes hypersensitive to oxidative stress^12^. Although an analogous manganese uptake system is present in GAS, the observation that loss of a zinc-efflux pump leads to decreased survival^10^ suggests that the zinc ions may also exert a direct antimicrobial effect within the cytoplasm of the bacteria. Therefore, to investigate the mechanism by which zinc mediates bacterial toxicity, we have employed the clinically-relevant, globally-disseminated M1T1 strain of GAS. Our findings indicate that elevated zinc disrupts virulence through the reduction of hyaluronic acid capsule biosynthesis. Additionally, zinc inhibits key enzymes of glucose catabolism, leading to a shift in expression of central carbon catabolic pathways.

## Results

### Zinc inhibition of the growth of GAS with glucose as carbon source

**Figure 1:**
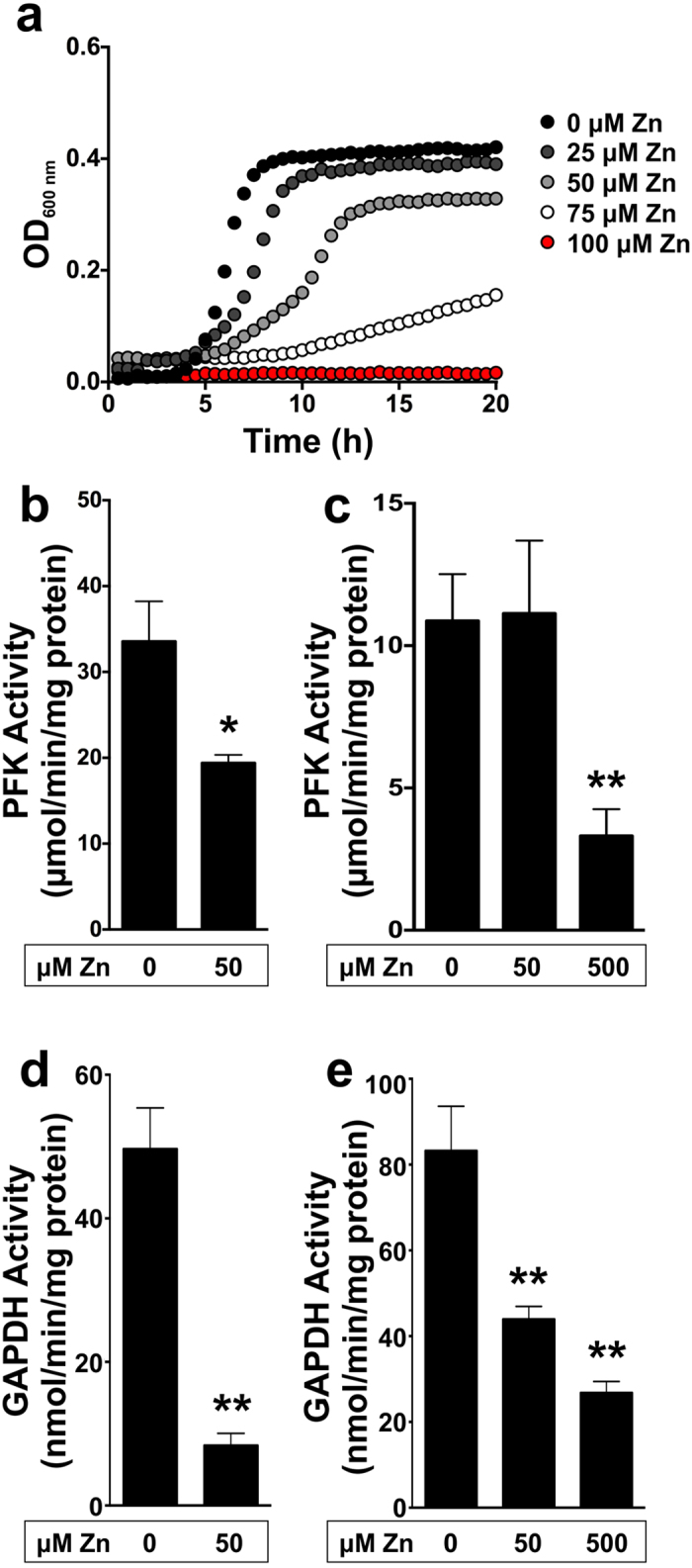
Growth of Group A *Streptococcus* in glucose and zinc results in inhibition of glycolytic enzymes. (**a**) Growth curve analysis of GAS 5448 WT in BHP-glucose supplemented with 0 (black circles), 25 (dark grey circles), 50 (light grey circles), 75 (white circles), 100 (red circles) μM zinc. Graph is a representative of three independent experiments. (**b**) PFK activity assay performed on GAS cell-free extracts of cells grown in BHP-glucose in the absence and presence of 50 μM zinc. Unpaired, 2-tailed t-tests were performed; *P < 0.05. (**c**) PFK activity assay performed on GAS cell-free extracts of cells grown in BHP-glucose. Cell-free extracts were incubated with 50–500 μM zinc for 10 min prior to the assay. Unpaired, 2-tailed t-tests were performed 0 vs. 500 μM zinc; **P < 0.01. (**d**) GAPDH activity assay performed on GAS cell-free extracts of cells grown in BHP-glucose in the absence and presence of 50 μM zinc. Unpaired, 2-tailed t-tests were performed; **P < 0.01. (**e**) GAPDH activity assay performed on GAS cell-free extracts of cells grown in BHP-glucose. Cell-free extracts were incubated with 50-500 μM zinc for 10 min prior to the assay. Unpaired, 2-tailed t-tests were performed on 0 vs. 50 μM zinc, and 0 vs. 500 μM zinc; **P < 0.01. Samples were collected during exponential phase of growth and error bars are indicative of the standard deviation of three independent experiments.

Studies of bacterial pathogens often make use of a complex growth medium where phenotypic and physiological effects of supplemented compounds such as transition metal ions are dampened^13^,^14^. Therefore, to overcome these problems, we designed a medium, BHP-glucose, with reduced nutrients compared to THY to allow for maximum utilization of the carbon source under investigation. With increasing concentrations of zinc, the growth rate and biomass formed by the GAS strain M1T1 isolate 5448 is reduced (Fig. 1a). At a zinc concentration of 100 μM in BHP-glucose medium, growth of GAS was inhibited. To achieve the same level of inhibition in complex THY medium, a concentration of 1.5 mM is required^10^.

Previous studies have suggested that zinc can inhibit specific enzymes in the glycolysis (Embden-Meyerhof) pathway^15^. Thus, we measured the enzymatic activity of phosphofructokinase (PFK) and Glyceraldehyde-3-phosphate dehydrogenase (GAPDH) under varying zinc concentrations during growth of GAS, and *in vitro* following addition of zinc to cell free extracts. The activity of PFK was significantly impaired in cells grown in the presence of 50 μM zinc compared to cells grown without zinc supplementation (*P* < 0.05, Fig. 1b). Furthermore, the addition of 500 μM zinc to cell free extracts reduced enzyme activity by 3-fold (*P* < 0.01, Fig. 1c). The activity of GAPDH was even more susceptible to zinc; in cells grown in the presence of 50 μM zinc there was 5-fold less activity compared to cells grown without addition of zinc (*P* < 0.01, Fig. 1d). Addition of zinc to cell free extracts also dramatically inhibited GAPDH activity (Fig. 1e). Therefore, our results are consistent with the hypothesis that zinc may exert toxicity towards GAS by inhibiting glycolysis, resulting in impaired growth. To confirm that the zinc inhibition observed was not due to a zinc-induced inability to import glucose, measurements of total glucose was undertaken at the start-, mid- and end-point of cells grown in BHP-glucose with increasing zinc concentrations. The results indicate that zinc does not inhibit glucose uptake in GAS (Supplementary Fig. 1), and the impaired growth was clearly due to the inhibition of glycolysis.

### Changes in GAS gene expression in response to zinc stress

To determine the global response of GAS to zinc, a transcriptome analysis was performed comparing the gene expression profile of the wild-type 5448 strain grown in THY in the presence and absence of 1 mM zinc. THY medium was used in this analysis to provide a rich growth environment for GAS, thereby reducing dependence on a single carbon source. Under these conditions, the level of zinc chosen to achieve a comparable level of sub-inhibitory toxicity as observed in BHP-glucose was 1 mM, a concentration previously shown to be sub-inhibitory in THY medium^10^. A total of 56 genes exhibited increased expression in the presence of zinc and 17 genes showed reduced expression (Supplementary table 1). Amongst the up-regulated genes, the *lac.2* operon and the galactose phosphotransferase (PTS) gene, *lacF*, showed increased expression in the presence of zinc (Table 1). Quantitative reverse-transcriptase PCR (qPCR) was performed on GAS cells grown in BHP-glucose in the absence and presence of 50 μM zinc to validate the microarray data obtained using THY medium containing 1 mM zinc. The qPCR results confirmed that the *lac.2* operon genes were upregulated in the presence of zinc (Supplementary Fig. 2). These data suggested a switch from glucose metabolism in the presence of zinc, to utilization of galactose as an alternative carbon source. GAS lacks the enzymes of the Leloir pathway (for the conversion of D-galactose to UDP-galactose) but can utilize the tagatose-6-phosphate pathway to convert D-galactose to dihydroxyacetone phosphate (DHAP) and glyceraldehyde phosphate (GAP) to partially bypass glycolysis to generate pyruvate. The first step in the tagatose-6-phosphate is the conversion of D-galactose to galactose-6-phosphate and then to tagatose-6-phosphate by galactose-6-phosphate isomerase (LacAB.2). The tagatose-6-phosphate is then converted to tagatose-1,6-bisphosphate by tagatose-6-phosphate kinase (LacC.2) and then finally converted to DHAP and GAP by tagatose-bisphosphate aldolase (LacD.2) (Table 1).

### Galactose utilization circumvents the zinc inhibited glucose utilization pathway in GAS

**Figure 2:**
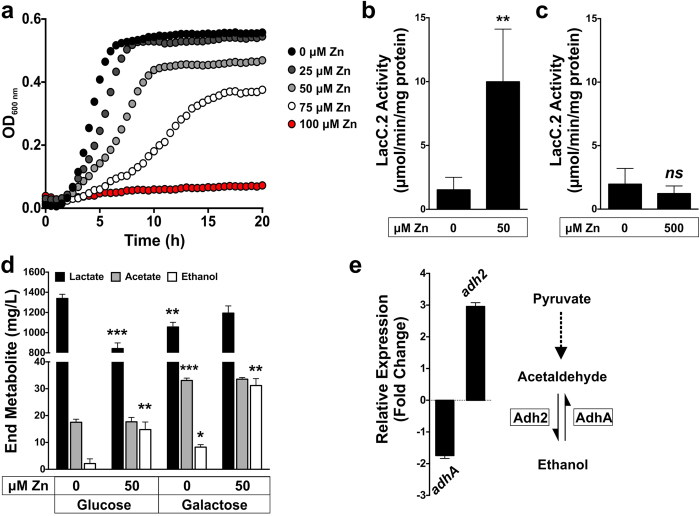
Zinc stress in Group A *Streptococcus* results in a shift in carbon metabolism. (**a**) Growth curve analysis of GAS 5448 WT in BHP-galactose supplemented with 0 (black circles), 25 (dark grey circles), 50 (light grey circles), 75 (white circles), 100 (red circles) μM zinc. Graph is a representative of three independent experiments. **(b)** Tagatose-6-phosphate kinase (LacC.2) activity assay performed on GAS cell-free extracts of cells grown in BHP-glucose in the absence and presence of 50 μM zinc. Unpaired, 2-tailed t-tests were performed; **P < 0.01. **(c)** Tagatose-6-phosphate kinase (LacC.2) activity assay performed on GAS cell-free extracts of cells grown in BHP-glucose. Cell-free extracts were incubated with 500 μM zinc for 10 min prior to the assay. Unpaired, 2-tailed t-tests were performed; *ns* = not statistically significant. Samples for enzyme activity were collected during exponential phase of growth and error bars are indicative of the standard deviation of three independent experiments. **(d)** End metabolite measurement of cells grown in either BHP-glucose or BHP-galactose in the presence and absence of 50 μM zinc. Cells were grown up to stationary phase in the described medium, supernatant collected, filtered and measured for L-lactate, acetate and ethanol. Graph is a representative of three independent experiments. Unpaired, 2-tailed t-tests were performed on: Lactate – glucose vs. galactose, glucose 0 vs. 25 μM zinc; Acetate – glucose vs. galactose; Ethanol – glucose vs. galactose, glucose 0 vs. 50 μM zinc, galactose 0 vs. 50 μM; ***P < 0.001, **P < 0.01, *P < 0.05. **(e)** Relative gene expression of the alcohol dehydrogenase genes, *adhA* and *adh2* (direction of reaction as indicated). Cells were grown up to mid-exponential phase in BHP-glucose in the presence and absence of 50 μM zinc, error bars are indicative of the standard deviation of 3 independent experiments. Relative gene expression was calculated using the 2^−ΔCT^ method, with *gyrA* as the reference gene, and results are represented as fold change of expression from growth in zinc compared to growth without zinc.

In view of the transcriptome data suggesting a potential switch in carbon metabolism, we investigated whether growth on galactose was less susceptible to zinc inhibition. Unlike the growth in BHP-glucose (Fig. 1a), GAS cells grown in BHP-galactose were less sensitive to zinc inhibition (Fig. 2a, Supplementary Fig. 3). The specific growth rates between GAS cells grown in BHP-glucose compared to BHP-galactose were similar, 0.48 and 0.47 h^−1^, respectively (*P* > 0.05; Supplementary Fig. 3b). However, the specific growth rate of GAS cells grown in BHP-glucose containing 75 μM zinc was 4-fold lower than GAS grown in BHP-galactose with 75 μM zinc, 0.02 compared to 0.08 h^−1^, respectively (*P* < 0.0001; Supplementary Fig. 3b). The same growth analyses were performed with other transition metal ions and no differential effect was observed between their sensitivities when grown in BHP-glucose compared to BHP-galactose (Supplementary Fig. 4), indicating that the growth inhibition in glucose is zinc-specific. Furthermore, the concentrations required for any minimal growth inhibition of GAS for all other metals tested was substantially higher than the concentration required for zinc-mediated growth inhibition.

Since transcription of the *lac.2* operon genes were increased in the presence of zinc, we proceeded to measure the enzymatic activity of tagatose-6-phosphate kinase (LacC.2) under varying zinc concentrations during growth of GAS, and *in vitro* following addition of zinc to cell free extracts. The activity of LacC.2 was significantly increased in cells grown in the presence of 50 μM zinc compared to cells grown without zinc supplementation (*P* < 0.01, Fig. 2b). However, unlike the PFK and GAPDH enzymes, the incubation of cell free extracts with 500 μM zinc did not significantly change the enzyme activity of LacC.2 (*P* > 0.05, Fig. 2c). These combined data suggest that the tagatose-6-phosphate pathway may be more resistant to high levels of zinc, and is upregulated when such conditions are encountered.

Previous studies have identified that GAS cells grown on different carbon sources result in different end product metabolites^16^. Therefore, we measured the metabolites produced by GAS during growth in BHP-glucose and BHP-galactose in the absence and presence of 50 μM zinc. In general, we found that growth in galactose resulted in decreased lactate (*P* < 0.01) and increased acetate (*P* < 0.001) and ethanol (*P* < 0.05) formation compared to growth in glucose (Fig. 2d). Growth in the presence of zinc resulted in a further increase of ethanol formation regardless of the carbon source (*P* < 0.01) and also a decrease in lactate formation (*P* < 0.001) when grown in glucose (Fig. 2d). This result indicates that the presence of zinc causes a shift in carbon source utilization and consequently, a shift in the end product metabolites. This was further supported by qPCR of the acetaldehyde/alcohol dehydrogenase gene (*adh2*), which converts acetyl-CoA to ethanol via acetaldehyde (Fig. 2e). These data show that growth in the presence of zinc results in the 3-fold up-regulation of *adh2*. In contrast the *adhA* gene that encodes a dehydrogenase involved in the oxidation of ethanol exhibited a 2-fold down-regulation (Fig. 2e).

### Zinc toxicity in GAS results in decreased hyaluronic acid capsule production

**Figure 3:**
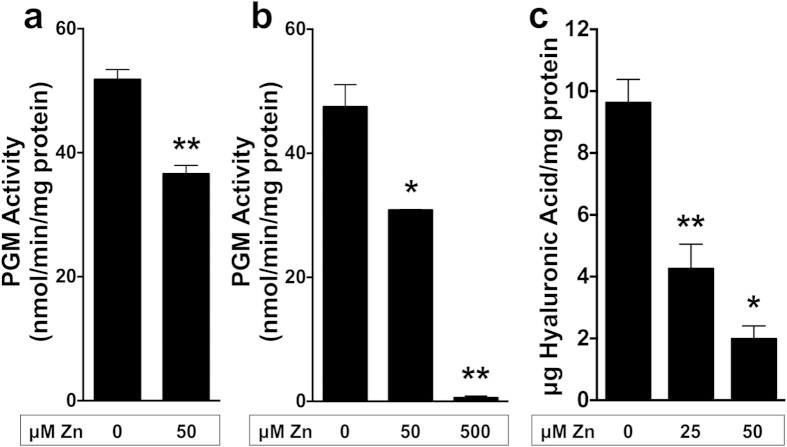
Growth of Group A *Streptococcus* in glucose and zinc results in decreased hyaluronic acid capsule. (**a**) PGM activity assay performed on GAS cell-free extracts of cells grown in BHP-glucose in the absence and presence of 50 μM zinc. Unpaired, 2-tailed t-tests were performed; **P < 0.01. (**b**) PGM activity assay performed on GAS cell-free extracts of cells grown in BHP-glucose. Cell-free extracts were incubated with 50–500 μM zinc for 10 min prior to the assay. Unpaired, 2-tailed t-tests were performed on 0 vs. 50 μM zinc, and 0 vs. 500 μM zinc; *P < 0.05, **P < 0.01. (**c**) Total hyaluronic acid measured on GAS cells grown in BHP-glucose in the absence and presence of 25 and 50 μM zinc. Unpaired, 2-tailed t-tests were performed on 0 vs. 25 μM zinc, and 0 vs. 50 μM zinc; **P < 0.01, *P < 0.05. All samples were collected during exponential phase of growth and error bars are indicative of the standard deviation of three independent experiments.

When glucose enters the cell, it can be used for a variety of biochemical processes, including nucleotide sugar metabolism. The first enzyme in this pathway, phosphoglucomutase (PGM), has also been identified as an enzyme susceptible to inhibition by zinc^17^. PGM converts glucose-6-phosphate to glucose-1-phosphate, which is then converted to UDP-glucose and UDP-glucuronic acid, precursors of the hyaluronic acid capsule of GAS. To determine if PGM in GAS is inhibited by zinc, we measured its enzymatic activity in the presence and absence of zinc. PGM activity was significantly reduced in GAS grown in the presence of 50 μM zinc (*P* < 0.01, Fig. 3a).Furthermore, when cell-free extracts of GAS cells grown in the absence of zinc were incubated with zinc 10 min prior to the assay, enzymatic activity was also significantly reduced *in vitro* with the addition of 50 and 500 μM zinc (*P* < 0.05 and *P* < 0.01, respectively; Fig. 3b). This result suggests that the decrease in PGM activity was not due to the secondary effects caused by reduced growth in the presence of zinc. We additionally assessed total hyaluronic acid content to determine if growth in zinc results in decreased hyaluronic acid capsule. Hyaluronic acid content decreased approximately 2-fold in cells grown in 25 μM zinc (*P* < 0.01), and further decreased by 5-fold in cells grown in 50 μM zinc (*P* < 0.05) (Fig. 3c). This result correlates with the decrease in PGM activity and suggests zinc toxicity results in reduction of hyaluronic acid capsule in GAS.

## Discussion

GAS is a host-adapted obligate human pathogen responsible for a wide spectrum of diseases. GAS can colonize the skin and throat asymptomatically or cause mild superficial infections such as impetigo and pharyngitis. GAS can also penetrate into deeper tissues resulting in severe invasive diseases such as septicaemia, necrotizing fasciitis, and streptococcal toxic shock-like syndrome^18^. GAS is estimated to cause more than 18 million cases of severe disease and over 500,000 human deaths per year^18^.

Zinc has recently been shown to be released by immune cells to combat intracellular pathogens including GAS^10^, however, the mechanism of zinc toxicity has not been previously described. Zinc is a critical metal ion for the nutrition and growth of all living organisms and it is the most abundant transition metal ion, being found in approximately 6% of proteins in bacteria^19^. Zinc within bacteria is allocated to enzymes and other proteins but the intracellular concentration of free zinc is usually kept very low and tightly regulated, as a consequence of zinc homeostasis^20^. GAS possesses two zinc uptake systems, AdcABC and Lsp (or AdcAII), regulated by AdcR^21^,^22^ that respond to zinc limitation. GAS also expresses a zinc efflux pump, CzcD, which is regulated by the GAS CzcD transcriptional activator, GczA^10^. By tightly controlling intracellular zinc, GAS should avoid zinc-mediated disruption of cell function. We have previously shown that deletion of the zinc efflux system in GAS caused a severe growth defect when challenged with zinc, elevated susceptibility to human neutrophil killing and loss of virulence in the murine model of invasive disease^10^. This suggests that high levels of zinc are toxic to GAS and that this is a condition encountered upon phagocytosis of GAS by immune cells. Here we have shown that zinc affects growth of GAS by decreasing its ability to use glucose as a carbon source as a consequence of the inhibition of two key enzymes of glycolysis, PFK and GAPDH (Fig. 1). This effect was exerted both at the level of enzyme function (Fig. 1b–e), and gene expression (Supplementary Fig. 5). In corroboration with our findings, excess zinc has previously been shown to inhibit glycolysis and glucose metabolism in various oral streptococcal species^23^,^24^.

Glucose is metabolized in GAS via the glycolytic pathway since it lacks the Entner-Doudoroff pathway^25^. The response of GAS to zinc appears to involve a switch in carbon metabolism to favor galactose utilization. We found that zinc toxicity in GAS is less pronounced when grown on galactose compared to glucose, and that this is a zinc-specific effect (Supplementary Fig. 3 and 4). Transcriptome data revealed that under zinc stress, the galactose/tagatose-6-phosphate metabolism is induced (Table 1, Supplementary Fig. 2). Furthermore, we confirmed that the LacC.2 activity and the *lacC.2* gene expression are increased when GAS is grown in the presence of zinc (Fig. 2b; Supplementary Fig. 2). Interestingly, a microarray study in *E. coli* comparing gene expression profiles of the cells grown in the absence and presence of N,N,N’,N’-tetrakis(2-pyridylmethyl)ethane-1,2-diamine (TPEN), a zinc chelator, identified the down-regulation of genes in the galactose/tagatose-6-phosphate metabolism during zinc starvation^26^. This further suggests a link between zinc homeostasis and central carbon metabolism. GAS possesses two *lac* operons (*lac*.1 and *lac*.2). Whilst the *lac*.1 operon is not directly involved in galactose metabolism but appears to have a regulatory role, the *lac*.2 operon is inducible and required for growth on galactose^27^,^28^,^29^. The enzymes in the galactose/tagatose-6-phosphate pathway convert imported galactose into metabolites that feed into glycolysis. It is also notable that at mucosal surfaces such as the airway epithelial and nasopharynx, a common niche for GAS, glucose concentration is low but galactose is readily available from mucins^30^,^31^. Furthermore, the membranes at these mucosal surfaces are sites of concentrated exchangeable zinc pools^8^,^32^. Therefore, with the abundance of zinc at mucosal sites, GAS may upregulate galactose metabolism and bypass the zinc inhibition of glucose utilization, resulting in the utilization of a carbon source readily available from the host.

**Figure 4:**
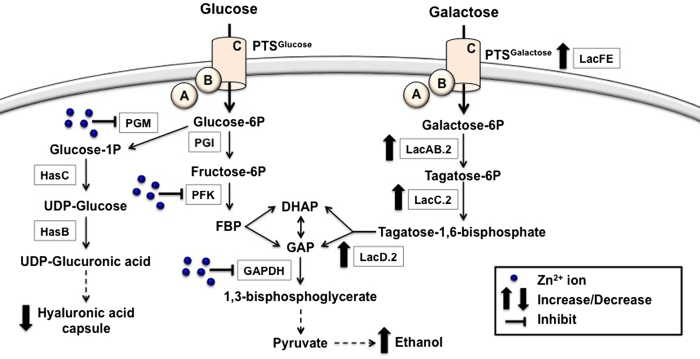
Proposed model demonstrating the effects of zinc on the physiology of GAS. Zinc stress results in inhibition of enzymes involved in glycolysis and capsule biosynthesis. As a result, the adaptive response of GAS to zinc stress is to switch from glucose to galactose utilization. PGI = phosphoglucose-6 isomerase; PFK = phosphofructokinase; GAPDH = glyceraldehyde-3-phosphate dehydrogenase; PGM = phosphoglucomutase; FBP = fructose-1,6,-bisphosphate; DHAP = dihydroxyacetone phosphate; GAP = glyceraldehyde-3-phosphate; PGM = phosphoglucomutase; HasC = UTP-glucose-1-phosphate uridylyltransferase; Has B = UDP-glucose-6 dehydrogenase. Zinc ions depicted by blue circles.

Studies have demonstrated that a shift from glucose to galactose utilization also results in a shift from homolactic fermentation (mainly lactate production) to mixed-acid fermentation (acetate, ethanol and formate production)^16^. Therefore, not surprisingly, our results show a shift in end-product metabolites resulting in increased ethanol production in the presence of zinc (Fig. 2d). Ethanol production in GAS can occur under aerobiosis by the pyruvate dehydrogenase complex or under microaerobic and anaerobic conditions by the oxygen-sensitive pyruvate formate lyase^25^. The host tissue environment favors mixed-acid fermentation due to the limited free and readily-fermentable sugars such as glucose, the abundance of glycoproteins with O and N-linked glycans that contain monosaccharides such as galactose and the hypoxic nature of deep tissue sites^31^. Combined, these observations have allowed us to build a model for zinc toxicity in GAS (Fig. 4) and suggest that zinc can act as a signal that regulates carbon metabolism and virulence in GAS.

Another enzyme that is inhibited by zinc is PGM. Elevated zinc levels led to a decrease in hyaluronic acid capsule formation. Glucose 1-P, the product of PGM, is required to generate UDP-glucose that forms the building blocks of hyaluronic acid, the capsular polysaccharide of GAS^33^,^34^. It has been shown that in GAS, growth on galactose instead of glucose results in decreased hyaluronic acid formation due to the reduction of UDP-sugar precursors^16^,^35^. Furthermore, deletion of PGM resulted in reduced capsule in *Streptococcus iniae* and decreased virulence of both *S. iniae* and *S. pneumoniae*^36^,^37^. It is well established that the hyaluronic acid capsule is associated with GAS virulence and its ability to resist phagocytic killing^38^,^39^,^40^. For example, an M1 GAS strain with a four-fold increase in capsule production had increased ability to disseminate into tissues, survive in human blood and resist neutrophil killing^41^. Likewise, GAS M-types that produces lower levels of capsule have reduced capacities to resist phagocyte killing^42^,^43^,^44^,^45^. Therefore, it is not surprising that in our previous study, deletion of the zinc efflux transporter CzcD, resulted in decreased virulence in the murine model of invasive disease^10^.

Our observations raise the question of the infection sites and timing during GAS infection in which zinc sequestration by the host plays a role in defense against infection versus the use of zinc by the host as an antimicrobial agent. It has been shown that a GAS *lsp* mutant that lacks the high affinity zinc acquisition system is attenuated in a murine subcutaneous ulcer model of infection, indicating that under these conditions zinc acquisition is critical for GAS infection^22^. Such a phenomenon may be explained through the sequestration of zinc by calprotectin released from neutrophils to impede microbial growth. Zinc sequestration as an antimicrobial strategy has been observed in the gut during infection with *Salmonella enterica* and *Helicobacter pylori*^46^,^47^. Our recent observations suggest that zinc plays a role in neutrophil killing and under those conditions, intracellular calprotectin may not play an antimicrobial role against GAS^10^. Furthermore, mucosal membranes containing secretory cells such as the airway epithelial and nasopharynx, common niches of GAS colonization, are sites of concentrated exchangeable zinc pools^8^,^32^. Combined, these observations support the hypothesis that zinc plays a central role in controlling bacterial infections through both sequestration and antimicrobial activity.

## Methods

### Bacterial strains and growth conditions

The *S. pyogenes* M1T1 clinical isolate 5448^48^ were routinely grown on 5% horse blood agar or statically in liquid cultures at 37 °C in Todd Hewitt broth supplemented with 1% yeast extract (THY). For physiological and enzymatic assays, cells were grown up in 1% brain heart infusion broth supplemented with 0.5% peptone (BHP) with the addition of either 1% D-glucose or D-galactose (BHP-glucose, BHP-galactose, respectively). When growing cultures in BHP overnight for subculturing the following day, 0.2% (w/v, final concentration) sodium bicarbonate was added prior to the inoculation of bacteria.

### Transcriptome analysis

M1T1 GAS 5448 was grown up in THY in the absence or presence of 1 mM zinc to mid-exponential phase (optical density ~0.6). Glass slide DNA microarrays were obtained from the Pathogen Functional Genomics Research Center (sponsored by the U.S. National Institute of Allergy and Infectious Diseases) at the J. Craig Venter Institute (Rockville, MD, USA). All probes representing the GAS M1T1 strain MGAS5005 ORFs were used for analysis. Total RNA was isolated using the RNeasy mini kit (Qiagen) and Cy3-labeled cDNA generated using the Fairplay microarray labelling kit (Stratagene). Single-colour hybridizations were performed according to the supplier’s instructions (http://pfgrc.jcvi.org/index.php/microarray/protocols.html). Slides were scanned using a GenePix 4000B scanner (Axon Instruments) and analysed using GenePixPro 4.0 software (Axon Instruments). Data from each microarray has undergone strict threshold and normalization as described^49^, and statistical analysis was performed using the MultiExperiment Viewer component of the TM4 microarray software suite^50^. Microarray data have been deposited in the Gene Expression Omnibus database (http://www.ncbi.nlm.nih.gov/geo) (accession no. GSE63436).

### Quantitative gene expression studies

RNA was isolated from 5 ml of GAS cells harvested under the desired growth phase in accordance with the RNeasy Mini kit (Qiagen) with the additional mechanical lysis step in lysing matrix B tubes (MP Biomedicals). The isolated RNA was DNase treated using the RNase-Free DNase set (Qiagen) and quantified using a Nanodrop instrument (Thermo Scientific). 1 μg of RNA was converted to cDNA using SuperScriptIII first-strand synthesis system for RT-PCR (Invitrogen). Real-time reverse transcriptase PCR was performed on the 1:5 diluted cDNA using the primers described in supplementary Table 1. The PCR reaction was performed using SYBR Green Master Mix (Applied Biosystems) according to manufacturer’s instructions. The reaction was performed in a ViiA7 real-time PCR system (Life Technologies), using the following conditions: 95 °C for 10 min, 40 cycles of 95 °C for 15 s, and 60 °C for 1 min, and a final dissociation cycle of 95 °C for 2 min, 60 °C for 15 s, and 95 °C for 15 s. All data was analyzed using the ViiA7 software (Life Technologies). Relative gene expression was calculated using the 2^−ΔCT^ method with *gyrA* as the reference gene.

### Growth analysis

Overnight cultures in BHP were diluted to OD_600_ ~ 0.05 in fresh BHP-glucose or BHP-galactose (in the presence of 0-100 μM zinc). The cells were statically grown in a 96-well microtitre plate and OD_600_ measured half-hourly using the FLUOstar OPTIMA (BMG Labtech) plate reader.

### Carbon metabolite assays

GAS cell cultures were grown in either BHP-Glucose or BHP-Galactose with or without the addition of 50 μM zinc (a sub-inhibitory zinc concentration). Samples (2 ml) were collected at start-, middle- and end-exponential and stationary phase for metabolite analysis. These samples were pelleted by centrifugation (5000 *g*, 5 min). The resulting supernatant was filter sterilised and used to assay metabolite concentrations using the D-glucose, L-lactic acid, acetic acid and ethanol determination kits according following manufacturer’s instructions (Megazyme).

### Enzyme activity assays

Cell free extracts were prepared from 20 ml GAS cultures grown to mid-exponential phase in either BHP-glucose or BHP-galactose with or without the addition of 50 μM zinc (a sub-inhibitory zinc concentration). GAS cells were pelleted, and the pellets resuspended and lysed in 0.5 ml of the assay buffer using the lysing matrix B (MP Biomedicals). The resultant supernatant was then used in the enzyme assay as follows: Glyceraldehyde-3-phosphate dehydrogenase (GAPDH) activity was performed as previously described^51^. Tagatose-6-phosphate kinase (LacC.2) activity was performed as previously described^52^. Phosphofructokinase (PFK) and phophoglucomutase (PGM) activities were measured according to the manufacturer’s instructions (BioVision and Sigma, respectively) via a coupled assay linked to the generation of NADH. Enzyme activity was normalized to total protein (mg) content by bicinchoninic acid (BCA) assay (Sigma).

### Hyaluronic acid measurement

GAS cell cultures were grown in BHP-Glucose with or without the addition of 25 and 50 μM zinc until OD_600_ - 0.4, and samples prepared as previously described^34^. Total hyaluronic acid content was measured using the hyaluronic acid (HA) test kit according to manufacturer’s instructions (Corgenix), and normalized to total protein (mg) content by BCA assay (Sigma).

### Statistical analysis

Differences in enzymatic activity, total metabolite and relative gene expression were analyzed using the 2-tailed *t* test (GraphPad, Prism 5).

## Additional Information

**How to cite this article**: Ong, C.- Y. *et al.* Zinc disrupts central carbon metabolism and capsule biosynthesis in *Streptococcus pyogenes.* *Sci. Rep.* **5**, 10799; doi: 10.1038/srep10799 (2015).

## Supplementary Material

Supplementary Information

## Figures and Tables

**Table 1:** Table of genes up-regulated by zinc involved in galactose transport and utilization.

**Locus**	**Gene/Locus**	**Function**	**Zn+/Zn-**	***P*****-Value**	***q*****-Value**
***Galactose transport***					
M5005_Spy1633	*lacE*	PTS system, lactose-specific IIBC component	2.792	0.000	0.028
M5005_Spy1634	*lacF*	PTS system, lactose-specific IIA component	2.754	0.000	0.031
					
***Enzymes involved in Tagatose-6-P/Galactose-6-P metabolism***					
M5005_Spy1635	*lacD.2*	Tagatose-bisphosphate aldolase	2.609	0.000	0.030
M5005_Spy1636	*lacC.2*	Tagatose-6-phosphate kinase	2.570	0.000	0.034
M5005_Spy1637	*lacB.2*	Galactose-6-phosphate isomerase lacB subunit	2.700	0.000	0.030
M5005_Spy1638	*lacA.2*	Galactose-6-phosphate isomerase lacA subunit	2.331	0.001	0.048
M5005_Spy1639	*lacR.2*	Lactose phosphotransferase system repressor	2.016	0.003	0.119
