# Supplementary material for: Zinc disrupts central carbon metabolism and capsule biosynthesis in Streptococcus pyogenes
Source: Sci Rep. 2015 Jun 1;5:10799. doi: 10.1038/srep10799 (PMC4450579; doi:10.1038/srep10799)
Supplement: Supplementary Information [file srep10799-s1.pdf]

Zinc disrupts central carbon metabolism and capsule biosynthesis in *Streptococcus pyogenes*

Cheryl-lynn Y. Ong[1], Mark J. Walker[1,#] and Alastair G. McEwan[1,#]

[1]School of Chemistry and Molecular Biosciences and Australian Infectious Diseases Research Centre, The University of Queensland, St. Lucia 4072, Australia.

[#]**Contributed equally to this work**

For correspondence:

Professor Alastair McEwan, School of Chemistry and Molecular Biosciences, The University of Queensland, Cooper Road, St Lucia, QLD, 4072, Australia. Tel: 0061-7-3365-3477; Fax: 0061-7-3346-0512; E-mail: mcewan@uq.edu.au.

Key words: zinc, group A streptococcus, *Streptococcus pyogenes*, glycolysis, capsule, tagatose-6-phosphate pathway

Supplementary Information:

Supplementary Tables S1-S2

Supplementary Figures S1-S5

Supplementary Tables

Table S1 Table of genes regulated by zinc, organized by gene number

Locus	Gene/Locus	Function	*gczA*+/ Δ*gczA*	*P*-Value	*q*-Value
Upregulated by zinc					
M5005_Spy_0083	*rpoB*	DNA-directed RNA polymerase beta chain	2.290	0.001	0.050
M5005_Spy_0084	*rpoC*	DNA-directed RNA polymerase beta' chain	2.337	0.001	0.048
M5005_Spy_0087	*comYB*	ComG operon protein 2	2.052	0.003	0.109
M5005_Spy_0088	*comYC*	ComG operon protein 3	2.278	0.002	0.085
M5005_Spy_0089	*Spy0089*	ComG operon protein 4	2.267	0.001	0.059
M5005_Spy_0090	*Spy0090*	Hypothetical protein	2.266	0.001	0.060
M5005_Spy_0091	*comYD*	ComG operon protein 6	2.573	0.000	0.028
M5005_Spy_0092	*Spy0092*	ComG operon protein 6	2.381	0.001	0.047
M5005_Spy_0102	*Spy0102*	Single-strand DNA binding protein	2.853	0.002	0.085
M5005_Spy_0151	*Spy0151*	3-keto-L-gulonate-6-phosphate decarboxylase	2.028	0.004	0.123
M5005_Spy_0153	*araD*	L-ribulose-5-phosphate 4-epimerase	2.067	0.004	0.123
M5005_Spy_0157	*opuAA*	Glycine betaine transport ATP-binding protein	3.577	0.000	0.034
M5005_Spy_0158	*opuABC*	Glycine betaine transport system permease	3.361	0.001	0.048
M5005_Spy_0245	*nifU*	IscU protein	2.106	0.003	0.118
M5005_Spy_0246	*Spy0246*	ABC transporter-associated protein	2.145	0.003	0.110
M5005_Spy_0473	*Spy0473*	Multidrug resistance protein B	2.079	0.004	0.140
M5005_Spy_0653	*czcD*	Cobalt zinc cadmium resistance protein	28.799	0.000	0.005
M5005_Spy_0875	*Spy0875*	Sortase	3.047	0.002	0.073
M5005_Spy_0990	*dnaE*	DNA polymerase III alpha subunit	2.707	0.004	0.128
M5005_Spy_1021	*Spy1021*	Phage protein	2.597	0.003	0.112
M5005_Spy_1022	*Spy1022*	Portal protein	3.147	0.000	0.031
M5005_Spy_1472	*hit*	Bis(5'-nucleosyl)-tetraphosphatase	2.032	0.003	0.110
M5005_Spy_1629	*salX*	Lantibiotic transport ATP-binding protein	2.677	0.000	0.029
M5005_Spy_1630	*salB*	Serine (threonine) dehydratase	2.701	0.001	0.048
M5005_Spy_1631	*salA*	Lantibiotic salivaricin A	2.238	0.002	0.090
M5005_Spy_1632	*lacG*	6-phospho-beta-galactosidase	2.459	0.001	0.052
M5005_Spy_1633	*lacE*	PTS system, lactose-specific IIBC component	2.792	0.000	0.028
M5005_Spy_1634	*lacF*	PTS system, lactose-specific IIA component	2.754	0.000	0.031
M5005_Spy_1635	*lacD.2*	Tagatose-bisphosphate aldolase	2.609	0.000	0.030
M5005_Spy_1636	*lacC.2*	Tagatose-6-phosphate kinase	2.570	0.000	0.034
M5005_Spy_1637	*lacB.2*	Galactose-6-phosphate isomerase lacB subunit	2.700	0.000	0.030
M5005_Spy_1638	*lacA.2*	Galactose-6-phosphate isomerase lacA subunit	2.331	0.001	0.048
M5005_Spy_1639	*lacR.2*	Lactose phosphotransferase system repressor	2.016	0.003	0.119
M5005_Spy_1640	*Spy1640*	DNA-damage-inducible protein J	2.115	0.003	0.120
M5005_Spy_1641	*Spy1641*	Hypothetical cytosolic protein	2.380	0.001	0.060
M5005_Spy_1642	*Spy1642*	DNA integration/recombination/invertion	2.313	0.003	0.119
M5005_Spy_1643	*Spy1643*	DNA integration/recombination/invertion	2.379	0.001	0.047
M5005_Spy_1644	*Spy1644*	Hypothetical protein	3.024	0.000	0.034

M5005_Spy_1646	*rpsI*	SSU ribosomal protein S9P	3.467	0.001	0.051
M5005_Spy_1647	*rplM*	LSU ribosomal protein L13P	2.471	0.001	0.069
M5005_Spy_1648	*Spy1648*	Transcriptional regulator, Cro/CI family	2.373	0.001	0.049
M5005_Spy_1649	*Spy1649*	Hypothetical membrane spanning protein	2.317	0.001	0.050
M5005_Spy_1670	*Spy1670*	Oxidoreductase	3.826	0.000	0.015
M5005_Spy_1671	*Spy1671*	Transcriptional regulator, MarR family	2.773	0.000	0.012
M5005_Spy_1807	*argR2*	Arginine repressor	5.380	0.000	0.028
M5005_Spy_1808	*argS*	Arginyl-tRNA synthetase	2.492	0.001	0.049
M5005_Spy_1810	*Spy1810*	Hypothetical membrane spanning protein	2.557	0.001	0.048
M5005_Spy_1811	*Spy1811*	Hypothetical membrane spanning protein	2.546	0.001	0.048
M5005_Spy_1812	*Spy1812*	Hypothetical membrane spanning protein	3.042	0.000	0.028
M5005_Spy_1813	*aspS*	Aspartyl-tRNA synthetase	3.720	0.000	0.013
M5005_Spy_1814	*hisS*	Histidyl-tRNA synthetase	3.154	0.000	0.029
M5005_Spy_1815	*rpmF*	LSU ribosomal protein L32P	4.444	0.000	0.010
M5005_Spy_1816	*rpmG*	LSU ribosomal protein L33P	3.685	0.000	0.017
M5005_Spy_1817	*cadD*	Cadmium resistance protein	3.603	0.000	0.015
M5005_Spy_1818	*cadC*	Cadmium efflux system accessory protein	3.162	0.000	0.031
M5005_Spy_1819	*Spy1819*	Hypothetical phage protein	3.036	0.001	0.053
Downregulated by zinc					
M5005_Spy_0350	*Spy0350*	Hypothetical protein	-3.484	0.000	0.013
M5005_Spy_0351	*spyA*	C3 family ADP-ribosyltransferase	-2.353	0.001	0.201
M5005_Spy_0437	*vicX*	Zinc-dependent hydrolase	-2.045	0.001	0.175
M5005_Spy_0534	*Spy0534*	(R,R)-butanediol dehydrogenase	-2.045	0.004	0.312
M5005_Spy_0582	*atpC*	ATP synthase epsilon chain	-2.169	0.004	0.332
M5005_Spy_0583	*Spy0583*	Hypothetical membrane associated protein	-2.300	0.003	0.317
M5005_Spy_0584	*murA*	UDP-N-acetylglucosamine-1-carboxyvinyltransferase	-2.439	0.002	0.229
M5005_Spy_0585	*epuA*	EpuA protein	-2.469	0.001	0.146
M5005_Spy_0586	*endA*	DNA-entry nuclease	-2.336	0.001	0.125
M5005_Spy_0885	*Spy0885*	DNA topoisomerase I	-6.060	0.000	0.000
M5005_Spy_0886	*Spy0886*	Transcriptional regulator, LysR family	-3.690	0.000	0.005
M5005_Spy_1034	*Spy1034*	Phage protein	-3.021	0.000	0.032
M5005_Spy_1036	*ssb*	Phage single-strand DNA binding protein	-2.353	0.000	0.117
M5005_Spy_1276	*Spy1276*	Transcription regulator, crp family	-3.300	0.000	0.013
M5005_Spy_1293	*Spy1293*	Hypothetical protein	-2.667	0.001	0.116
M5005_Spy_1358	*nadE*	Nicotinate phosphoribosyltransferase	-2.203	0.004	0.322
M5005_Spy_1678	*Spy1678*	Thioredoxin	-2.119	0.003	0.333

Table S2 List of primers used in this study

Primer	Gene / Function	Sequence (5'-3')
Gene expression study		
gyrA-F	*gyrA* / housekeeping	GAAGTGATCCCTGGACCTGA
gyrA-R		CCCGACCTGTTTGAGTTGTT
adh2-F	*adh2* / alcohol dehydrogenase	GCTATCCGCGTCATCTGGAA
adh2-R		ACCAACTGCGTTACGTCCAT
adhA-F	*adhA* / acetaldehyde dehydrogenase	ATTGGTCATGGTGAAGCGCT
adhA-R		TGACCAAAATCGCCATGTGC
pgmA-F	*pgmA* / phosphoglucomutase	TGCTTTTGAGTCTGCACAGG
pgmA-R		AGAGTTCTGGTGTTGGACGA
gapN-F	*gapN* / glyceraldehyde-3-P dehydrogenase	CAACGTTGGCCTGAGTAACC
gapN-R		CTTGGCGGTAAGGATTCTGC
pfk-F	*pfk* / phosphofructokinase	TGGCCAAGATTTGTCACACG
pfk-R		GCTGAAGGTGTTATGAGCGG

Supplementary Figures

Supplementary Figure 1.

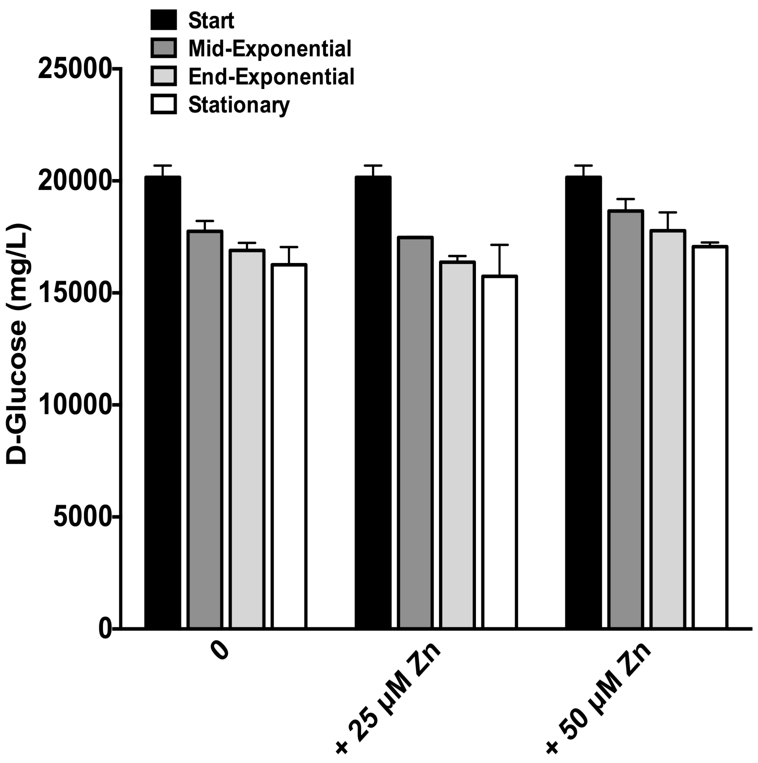

Supplementary Figure 1 Total D-glucose measurement of cells grown in BHP-glucose in the presence and absence of 25 and 50 µM zinc. Supernatant was collected, filtered and measured for D-glucose at different stages of growth. Graph is a representative of three independent experiments.

Supplementary Figure 2.

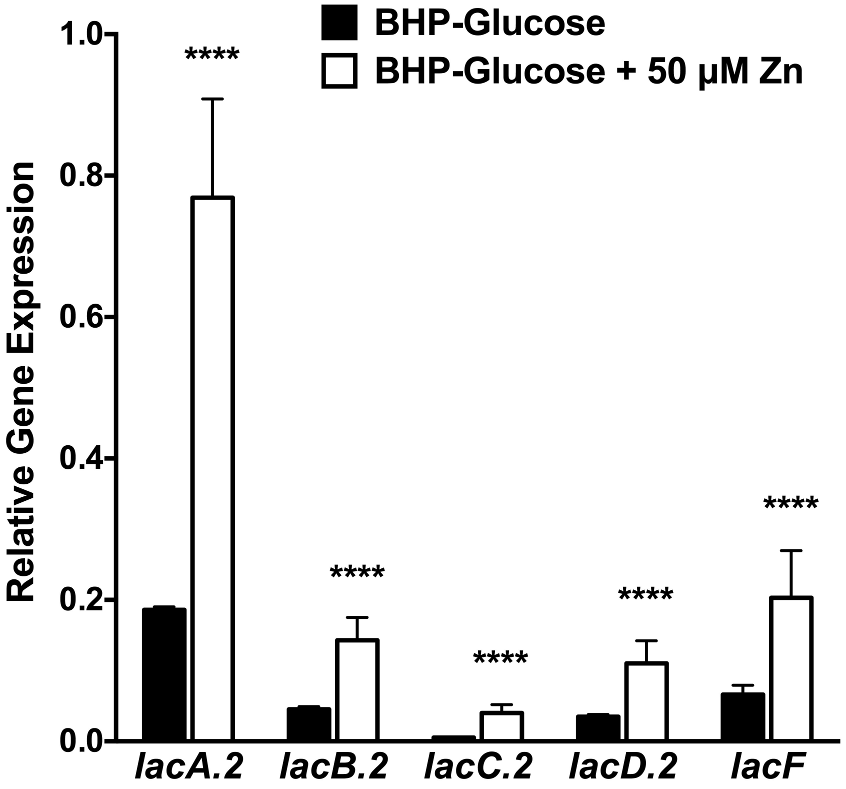

Supplementary Figure 2 Relative gene expression of the tagatose-6-phosphate metabolism genes, *lacF* (encodes D-galactose phosphotransferase), *lacD.2* (encodes tagatose-6-phosphate aldolase), *lacC.2* (encodes tagatose-6-phosphate kinase), *lacB.2* and *lacA.2* (encodes galactose-6-phosphate isomerase A/B subunits). Cells were grown up to mid-exponential phase in BHP-glucose in the presence and absence of 50 µM zinc, error bars are indicative of the standard deviation of 3 independent experiments. Relative gene expression was calculated using the $2^{-\Delta CT}$ method, with *proS* as the reference gene. Unpaired, 2-tailed *t* test was performed between relative gene expression of cells grown in BHP-glucose in the absence and presence of 50 µM zinc; *****P*<0.0001.

Supplementary Figure 3.

a

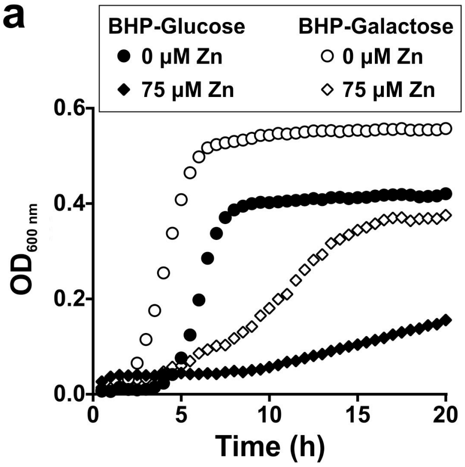

b

Growth Condition	Specific Growth Rate h^{-1} (S.D.)	
BHP-Glucose	0.4853 (±0.163)	
BHP-Galactose	0.4657 (± 0.134)	
BHP-Glucose + 75 µM Zn	0.0162 (±0.011)	****
BHP-Galactose + 75 µM Zn	0.0752 (±0.018)	

*S.D. - Standard deviation; **** P<0.0001*

Supplementary Figure 3 Growth curve and specific growth rate analysis of Group A *Streptococcus* grown in BHP-glucose, or BHP-galactose in the absence and presence of zinc. (a) Growth curve analysis in the absence and presence of 75 µM zinc. Graph is a representative of five independent experiments. **(b)** Table of the specific growth rates (h^{-1}) of GAS cells grown in the absence and presence of 75 µM zinc. Values shown are the mean specific growth rates of five independent experiments. Unpaired, 2-tailed *t* test was performed between growth rate of cells grown in BHP-glucose in the presence of 75 µM zinc and cells grown in BHP-galactose in the presence of 75 µM; ****P<0.0001.

Supplementary Figure 4.

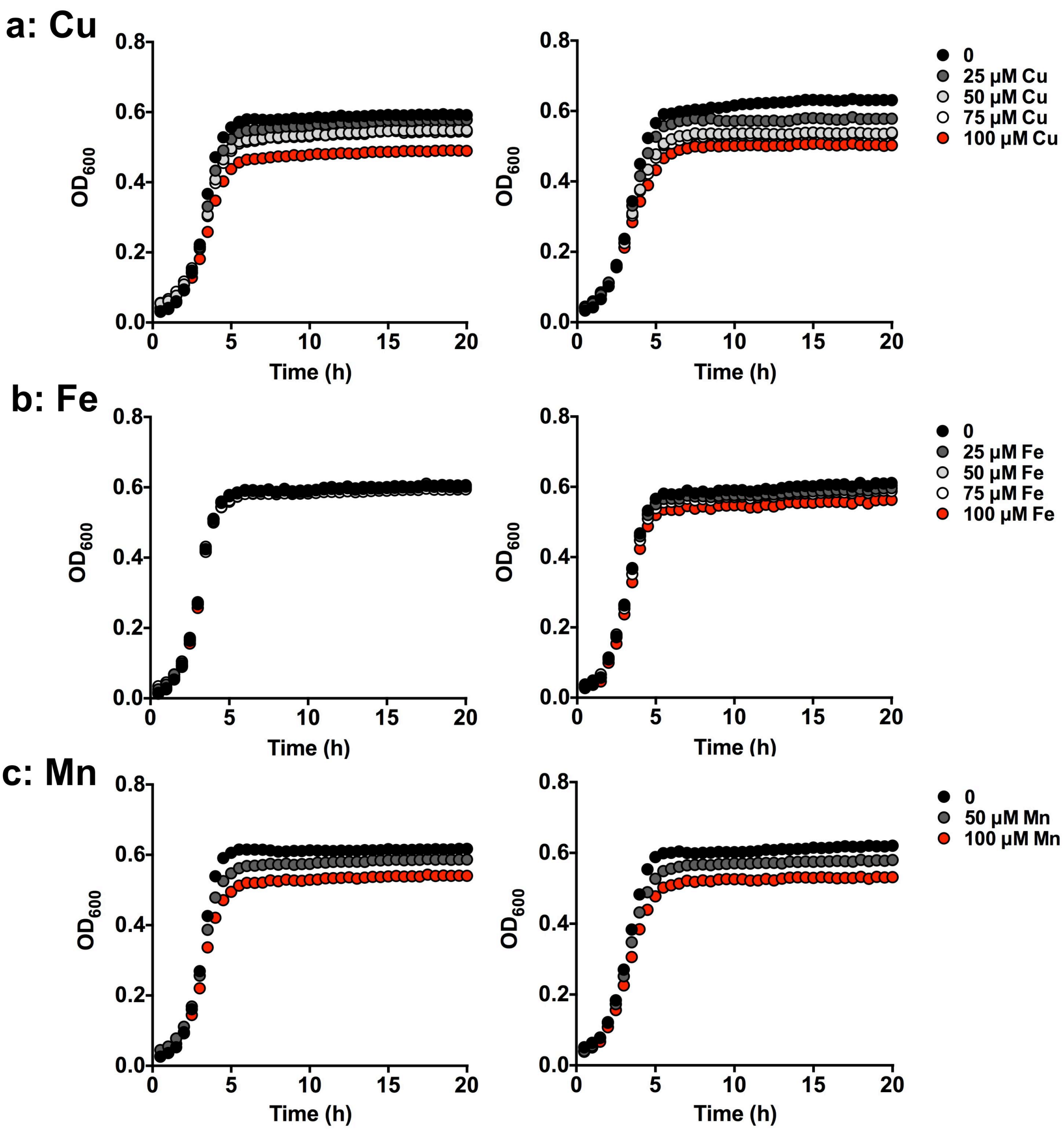

Supplementary Figure 4 Growth curve analysis of Group A *Streptococcus* grown in BHP-glucose, or BHP-galactose in the absence and presence of divalent metal ions. (a) copper, **(b)** iron and, **(c)** manganese. Graph is a representative of three independent experiments.

Supplementary Figure 5.

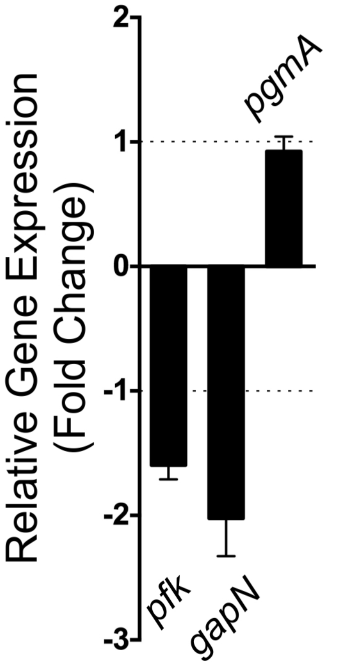

Supplementary Figure 5 Relative gene expression of the glycolytic genes, *pfk* (encodes phosphofructokinase), *gapN* (encodes glyceraldehyde-3-phosphate dehydrogenase) and *pgmA* (encodes phosphoglucomutase). Cells were grown up to mid-exponential phase in BHP-glucose in the presence and absence of 50 µM zinc, error bars are indicative of the standard deviation of 3 independent experiments. Relative gene expression was calculated using the $2^{-\Delta CT}$ method, with *gyrA* as the reference gene, and results are represented as fold change of expression from growth in zinc compared to growth without zinc.
